# Pneumococcal vaccination effectiveness (PCV13 and PPSV23) in individuals with and without reduced kidney function: a test-negative design study

**DOI:** 10.1093/ckj/sfae145

**Published:** 2024-05-08

**Authors:** Dustin Le, Alexander Chang, Morgan E Grams, Josef Coresh, Junichi Ishigami

**Affiliations:** Division of Nephrology, Department of Medicine, Johns Hopkins School of Medicine, Baltimore, MD, USA; Department of Epidemiology, Johns Hopkins Bloomberg School of Public Health, Baltimore, MD, USA; Departments of Nephrology and Population Health Sciences, Geisinger Health, Danville, PA, USA; Division of Precision Medicine, Department of Medicine, New York University, New York, NY, USA; Optimal Aging Institute, Department of Medicine, New York University, New York, NY, USA; Department of Epidemiology, Johns Hopkins Bloomberg School of Public Health, Baltimore, MD, USA

**Keywords:** chronic kidney disease, pneumococcal vaccination

## Abstract

**Background:**

*Streptococcus pneumoniae* vaccination effectiveness (VE) in individuals with reduced kidney function is unknown. We estimated pneumococcal conjugate vaccine (PCV13), pneumococcal polysaccharide vaccine (PPSV23), and combined PCV13 and PPSV23 effectiveness against pneumococcal disease in individuals with and without reduced estimated glomerular filtration rate (eGFR).

**Methods:**

All eligible individuals (case and controls) were adults (aged ≥18 years) hospitalized within the Geisinger Health System and required to have *S. pneumoniae* urinary antigen testing (i.e. test-negative design). Vaccination records were obtained from the electronic health record and statewide vaccination registry. After controlling for the probability of receiving a pneumococcal vaccine, we used multivariable logistic regression models to estimate the odds ratios (ORs) of vaccination between those who did and did not meet the *S. pneumoniae* case definition. VE was calculated as (1 – OR) × 100%.

**Results:**

There were 180 cases and 3889 controls (mean age 69 years, female 48%, white 97%, mean eGFR 71 mL/min/1.73 m^2^). The adjusted population PCV13 VE was 39% (95% CI 13%–58%), and combination PCV13 and PPSV23 was 39% (95% CI 12%–58%). PPSV23 VE was –3.7% (95% CI –57% to 32%). Stratified by eGFR, adjusted PCV13 VE was consistent in eGFR ≥60 [VE 38% (95% CI 2.9%–61%)] and 30–59 [VE 61% (95% CI 24%–80%)] without significant interaction. VE was not calculable for eGFR <30 due to small sample size.

**Conclusion:**

PCV13 vaccination was associated with reduced risk of *S. pneumoniae* hospitalization in individuals with a reduced eGFR (30–59 mL/min/1.73 m^2^).

KEY LEARNING POINTS **What was known:**

- *Streptococcus pneumoniae* vaccination is an effective strategy for reducing *S. pneumoniae* hospitalization and bacteremia in the general population.
- Patients with kidney disease, however, have decreased immune response to vaccination, and the impact on clinical outcomes is unknown.

**This study adds:**

- Pneumococcal conjugate vaccine (PCV13) conferred protection against *S. pneumoniae* hospitalization and bacteremia in individuals with reduced estimated glomerular filtration rate (eGFR 30–59 mL/min/1.73 m^2^).
- There was no clear evidence of pneumococcal polysaccharide vaccine (PPSV23) effectiveness against *S. pneumoniae* hospitalization or bacteremia.

**Potential impact:**

- Efforts to increase pneumococcal vaccination are needed to decrease rates of *S. pneumoniae* hospitalization and bacteremia.
- Individuals with reduced eGFR may particularly benefit from pneumococcal vaccination due to their high risk of infection.

## INTRODUCTION

Pneumonia is a major cause of hospitalization and death in the USA with annual medical costs >$10 billion in 2011. *Streptococcus pneumoniae* is the most common bacterial etiology [1], leading to 150 000 hospitalizations a year [2], and individuals with chronic kidney disease (CKD), which affects 37 million individuals in the USA, are at higher risk of pneumonia hospitalization [2, 3]. Therefore, vaccination should be an important strategy to reduce the burden of pneumococcal pneumonia in this population.

Current vaccination guidelines, however, are inconsistent for individuals with CKD. For example, the Advisory Committee on Immunization Practices (ACIP) at the Center for Disease Control recommends pneumococcal conjugate vaccine (PCV) for individuals with chronic renal failure without discussion of CKD stage [4]. The old 2012 Kidney Disease: Improving Global Outcomes (KDIGO) CKD guidelines recommend pneumococcal vaccination starting at severely decreased kidney function, defined as an estimated glomerular filtration rate (eGFR) of <30 mL/min/1.73 m^2^ [i.e. CKD stage 4, 5 and end-stage kidney disease (ESKD)] [5]. Furthermore, the new 2024 KDIGO guidelines do not recommend specific vaccination strategies [6]. To date, no guidelines offer specific recommendations for the vast majority of the CKD population (with eGFR 30–59, i.e. stage 3 CKD) who are also at increased risks of infection and hospitalization [7].

Part of this gap may be due to limited data linking pneumococcal vaccination to pneumococcal-specific clinical outcomes in individuals with non-dialysis CKD [8]. Previous observational studies of pneumococcal polysaccharide vaccine 23 (PPSV23) have shown modest all-cause survival benefit in ESKD [9], but no benefit was seen against pneumonia hospitalization in either CKD [10] or ESKD [11]. For pneumococcal conjugate vaccine 13 (PCV13), there are no CKD-specific data, but a recent study suggested vaccine benefit against pneumonia hospitalization may be diminished in individuals with chronic medical conditions including “chronic renal failure” [12]. In terms of serologic data, multiple studies in non-dialysis CKD have shown diminished immune response against vaccination [13] in a dose-dependent fashion with CKD stages [14], raising concerns about clinical benefit. This uncertainty likely hampers vaccination recommendations for individuals with CKD—especially those with mild to moderate CKD.

To address this knowledge gap, we characterized the vaccine effectiveness (VE) of PCV13 and PPSV23 against pneumococcal pneumonia among individuals with and without reduced eGFR using data from a large integrated US health system, the Geisinger Health System.

## MATERIALS AND METHODS

### Study population

We included all individual within Geisinger (a large regional health system in central and northeastern Pennsylvania) [15] age ≥18 years at hospitalization with pneumococcal urinary antigen testing. Individuals were excluded for either missing baseline creatinine data (previous 18 months) or history of ESKD/solid-organ transplant.

This study was approved by the Johns Hopkins University and Geisinger Medical Center Institutional Review Boards (IRB). The need for informed consent was waived by the IRB.

### Test-negative design

We estimated the effectiveness of pneumococcal vaccination using a test-negative design. In this study design, the study population consisted of all individuals who underwent urine pneumococcal antigen testing. Individuals who tested positive served as cases (going forward, we call these test-negative cases), and those who tested negative served as controls (test-negative controls). Because all test-negative cases and controls received testing, they presumably had a similar presentation warranting the same clinical test. Compared with a case–control study (where controls are randomly selected), this study design is considered to reduce disease misclassification, confounding by indication, and healthy vaccinee bias [16].

### Test-negative case and test-negative control definition

Given high specificity (97%) but lower sensitivity (74%) of pneumococcal urine antigen testing [17], we identified test-negative cases as hospitalizations with either (i) a positive urinary antigen test or (ii) negative urinary antigen test with positive culture (blood, urine or sputum) for *S. pneumoniae* or International Classification of Diseases (ICD) code for *S. pneumoniae*. There were zero cases with cerebrospinal fluid positive for *S. pneumoniae*. Test-negative controls had negative pneumococcal urine antigen testing and did not meet the test-negative case definition. Individuals could contribute multiple hospitalizations, but hospitalizations after case hospitalization were excluded.

### Vaccination status

Vaccination history was ascertained by electronic health record. Vaccinations within the Geisinger Health System are updated into the patient record, and since November 2011, there has been a bidirectional linkage with the Pennsylvania Statewide Immunization Information System [18]. Within these records, 18% of pneumococcal vaccinations were updated via patient report as “PCV13” or “PPSV23.” Receipt of vaccination was defined as >30 days prior to hospitalization.

We additionally report the yearly point prevalence of PCV13 and PPSV23 vaccination uptake among all adults aged ≥18 years. Individuals without a baseline creatinine in a calculated calendar year were excluded.

### Covariates

Age, sex, race and body mass index were abstracted from the electronic health record. Comorbid conditions were assessed using ICD, Tenth Revision, Clinical Modification (ICD-10-CM) codes (Supplementary data, Table S1) and recorded if there were two separate encounters prior to hospitalization. Smoking status was abstracted from self-report on admission as former, current or never smoker. Immunosuppressive medications at admission were noted (i.e. biologics, antimetabolites, corticosteroids, calcineurin and non-calcineurin inhibitors). Steroids equivalent to ≥20 mg of prednisone for ≥30 days were also defined as an immunosuppressive medication. Intravenous, inhaled and solution steroids were excluded. Covariates were updated for each hospitalization. Data were complete except for smoking. These 111 (of 4067) were addressed using multiple imputation.

We used the most recent outpatient creatinine measurement ≤18 months prior to hospitalization and the race-free 2021 CKD Epidemiology Collaboration equation to estimate eGFR [19]. Creatinine values from hospitalizations or emergency department evaluations were excluded to minimize measurements from acute kidney injury.

### Statistical analysis

Baseline characteristics between test-negative cases and test-negative controls were compared using Pearson χ^2^ or Fisher's exact test for categorical variables and t-tests for continuous variables.

Given the non-randomized, observational design and possible biases in vaccination (such as the healthy vaccinee bias) [20], we used inverse probability treatment weighting (IPTW). We calculated a propensity score of vaccination using multivariable logistic models regressed on age, sex, race (“white” or “not white”), body mass index, smoking status, eGFR, diabetes, myocardial infarction, stroke, congestive heart failure, peripheral vascular disease, dementia, pulmonary disease, rheumatologic disease, cancer, liver disease, immunosuppressive use, year of hospitalization and recent hospitalization (<1 year). The IPTW was calculated as W = [ZPr(*Z* = 1)]/*e* + [(1 – *Z*)Pr(*Z* = 0)]/(1 – *e*), where $e$ = propensity score of vaccination, $Z$ = vaccination status (*Z* = 1, vaccinated; *Z* = 0, not vaccinated), and Pr(*Z* = 1) versus Pr(*Z* = 0) was the overall probability of vaccination or no vaccination [21].

We calculated the odds ratios (ORs) of receiving pneumococcal vaccination between test-negative cases and controls using multivariable logistic regression models adjusted for the same covariates as above in addition to controlling for overall vaccine receipt via IPTW (i.e. doubly robust estimation) [22]. VE was calculated as (1 – OR) × 100 with 95% confidence intervals (95% CIs) [23]. Analysis was performed for the overall population and repeated by eGFR strata (≥60, 30–59 and <30 mL/min/1.73 m^2^ which correspond to mild, moderate and severe kidney disease, respectively).

We repeated doubly robust estimation for each group of PCV13, PPSV23, and combined PCV13 and PPSV23 with repeat hospitalizations per individual accounted for using clustered standard errors. We additionally tested for interaction by eGFR group and VE in individuals with eGFR ≥30 mL/min/1.73 m^2^.

We conducted multiple sensitivity analyses. First, given higher VE against bacteremic disease [24], we compared VE between bacteremic (defined by PCR testing) and non-bacteremic pneumococcal pneumonia with additional stratification by eGFR (≥60, 30–59 and <30 mL/min/1.73 m^2^). Second, we stratified analyses by years since last vaccination to examine waning immunity after vaccination (PCV13: no vaccine, <2 years, 2–4 years and >4 years; and PPSV23: no vaccine, <5 years, 5–10 years, 10–15 years and >15 years). Third, to increase specificity, we restricted controls to individuals who had a confirmed infection other than *S. pneumoniae* by positive PCR (blood, urine or sputum) (e.g. *Staphylococcus aureus*), urinary antigen test for *Legionella* bacterium, nasal swab for severe acute respiratory syndrome coronavirus 2 or ICD-10-CM code for non-*S. pneumoniae* (Supplementary data, Table S6). We then performed separate analyses with restriction of creatinine measurements to the previous 12 months, vaccination defined as ≥14 days prior to hospitalization and exclusion of cases defined by ICD code. Next, we included vaccination with the opposing vaccine in the propensity score model (i.e. history of PPSV23 vaccination in the PCV13 IPTW and history of PCV13 vaccination in the PPSV23 IPTW).

Significant test results were determined by two-tailed *P*-value < .05. Analyses were performed using R Core Team, Version 4.2.2 (R Foundation for Statistical Computing, Vienna, Austria) [25].

## RESULTS

### Study population

Among individuals aged ≥18 years between 1 January 2016 and 31 August 2021, there were 479 951 hospitalizations, and 5601 (1.2%) had urinary antigen testing. After exclusions, there were 4067 admissions with 180 test-negative cases and 3887 test-negative controls (Supplementary data, Fig. S1), with 10% of test-negative controls being repeat hospitalizations. Of the 180 test-negative cases, approximately 140+ had a positive urinary antigen test, 20+ had a positive blood, urine or sputum culture, and <10 had a (+) ICD code for *S. pneumoniae*.

### Baseline characteristics

In the study population, the average individual was age 69 ± 15 years, 52% were male, 97% were white and 78% had one or more medical condition. The most common chronic conditions included chronic respiratory disease (35%), eGFR <60 mL/min/1.73 m^2^ (35%) and diabetes (29%). Compared with test-negative controls, cases were similar in age and white race, but more likely to be female, a current smoker and vaccinated with PCV13 (Table 1). Cases and controls did not significantly differ by remaining comorbidities, immunosuppression use or baseline eGFR. When baseline characteristics were compared across eGFR strata (≥60, 30–59, <30 mL/min/1.73 m^2^), those with lower eGFR were more likely to be older, female and vaccinated (separately and in combination), and to have more chronic health conditions (Supplementary data, Table S2).

**Table 1:** Demographics: test-negative cases and controls with *Streptococcus pneumoniae*^a^.

Characteristic	All (*N* = 4067)	Test-negative cases (*N* = 180)	Test-negative controls (*N* = 3887)	*P*-value^b^
Age (years), mean (SD)	69 (15)	68 (15)	69 (16)	.24
Female	1941 (48)	111 (62)	1830 (47)	<.01
White	3950 (97)	174 (97)	3776 (97)	.91
Body mass index (kg/m^2^), mean (SD)	29 (9.3)	29 (8.7)	29 (9.2)	.75
Never smoker	1288 (32)	40 (22)	1248 (32)	<.01
Former smoker	2041 (50)	73 (41)	1968 (51)	
Current smoker	717 (18)	46 (26)	671 (17)	
PPSV23 vaccination	3154 (78)	136 (76)	3018 (78)	.58
PCV13 vaccination	2057 (51)	75 (42)	1982 (51)	.02
PPSV23 and PCV13 vaccination	1928 (47)	70 (39)	1858 (48)	.02
Recent hospitalization (<1 year)	2523 (62)	105 (58)	2418 (62)	.33
Diabetes mellitus	1166 (29)	48 (27)	1118 (29)	.60
Myocardial infarction	163 (4)	<10 (5)	154 (4.0)	.89
Stroke	351 (8.6)	21 (12)	330 (8.5)	.18
Congestive heart failure	845 (21)	37 (21)	808 (21)	.99
Peripheral vascular disease	384 (9.4)	19 (11)	365 (9.4)	.69
Dementia	49 (1.2)	<10 (2)	46 (1.2)	.75
Pulmonary	1407 (35)	67 (37)	1340 (35)	.50
Chronic obstructive pulmonary disease	1074 (26)	57 (32)	1017 (26)	.12
Rheumatologic	117 (2.9)	<10 (3)	111 (2.9)	.67
Cancer	546 (13)	23 (13)	523 (13.4)	.88
Liver	129 (3.2)	<10 (4)	122 (3.1)	.84
Peptic ulcer disease	15 (0.4)	<10 (2)	12 (0.3)	.06
Hemiplegia	17 (0.4)	0 (0.0)	17 (0.4)	.99
Immunosuppression use	170 (4.2)	10 (5.6)	160 (4.1)	.45
eGFR (mL/min/1.73 m^2^)^c^	71 (35–105)	71 (34–105)	71 (35–105)	.80
≥60	2612 (64)	117 (65)	2495 (64)	.66
≥30 and <60	1182 (29)	55 (31)	1127 (29)	
≥15 and <30	241 (6)	<10 (4)	233 (6)	
<15	32 (<1)	0 (0)	32 (<1)	
Hospitalization year				.20
2016	222 (5.5)	14 (7.8)	208 (5.4)	
2017	414 (10)	18 (10)	396 (10)	
2018	633 (16)	33 (18)	600 (15)	
2019	1158 (29)	47 (26)	1111 (29)	
2020	1120 (27)	54 (30)	1066 (27)	
2021	520 (13)	14 (8)	506 (13)	

Past medical history defined by ICD-10 codes.

a Data are presented as mean [standard deviation (SD)] or mean (%).

b Pearson χ^2^, Fisher's exact (*N* < 10), or t tests comparing cases vs controls.

c Race-free 2021 eGFR_Cr_ presented as mean (10th–90th percentile) mL/min/1.73 m^2^_._

### Prevalence of pneumococcal vaccination

**Figure 1:**
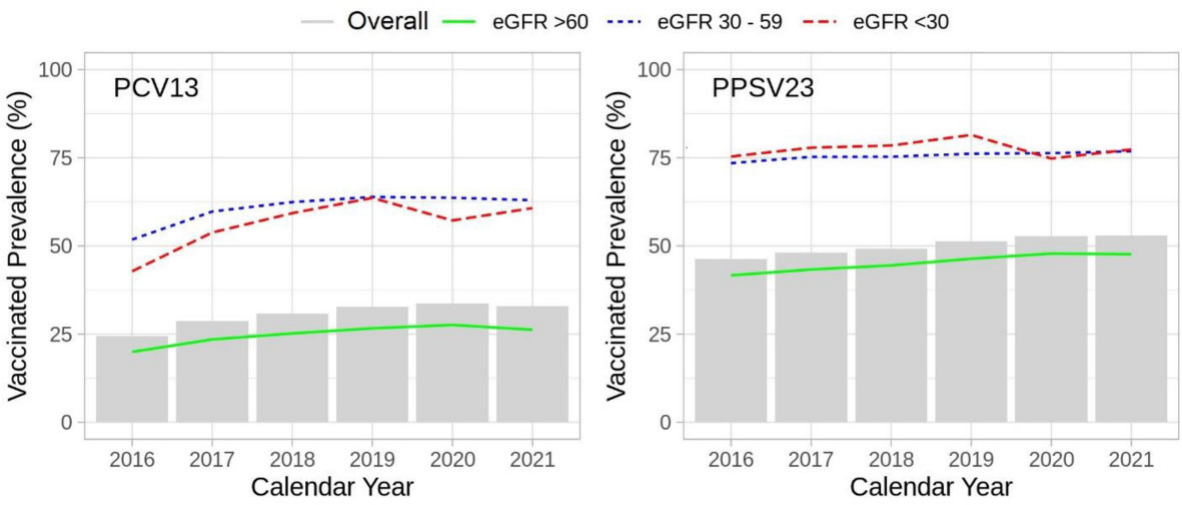
Crude PCV13 and PPSV23 vaccination prevalence among adults (≥18 years) in the Geisinger Health System from 2016 to 2021** **(*N* = 431 568). Pneumococcal vaccination uptake increased over time with individuals with decreased GFR having higher rates of vaccination.

The proportions having received PCV13, PPSV23 or both prior to hospitalization were 51%, 78% and 47%. These numbers were greater than the vaccination prevalence in the Geisinger Population (2016–21) (e.g. 32% for PCV13 and 52% for PPSV23) [Fig. 1 and Supplementary data, Fig. S2 (by age)], likely reflecting the study population who developed symptoms that warranted a pneumococcal antigen test.

Within the study population, median time since last PCV13 was 2.7 years (25th–75th percentile: 1.2–4 years), and PPSV23 was 2 years (25th–75th percentile: 0.6–5.8 years). Among those with both PCV13 and PPSV23, 103 individuals received both vaccinations within 30 days of each other, and the median difference between the two vaccinations was 6 years (25th–75th percentile: 2–10 years). Between those who did and did not receive pneumococcal vaccination, many characteristics such as age, sex, eGFR and medical comorbidities were significantly different by standardized mean differences >0.10, but these characteristics became similar after IPTW (Supplementary data, Fig. S3). Of note, 129 individuals received PCV13 without history of PPSV23, and thus, 94% (1928 of 2057) who received PCV13 also received PPSV23. For PPSV23, only 61% of individuals with PPSV23 had received PCV13.

### Population effectiveness of PCV13 and PPSV23

Forty-two percent of cases and 51% of controls were vaccinated with PCV13 (*P*-value = .02). In unadjusted analysis in the overall population, PCV13 vaccination was significantly associated with lower odds of pneumococcal diseases [VE 31% (95% CI 7%–49%), Table 2]. After adjustment, the VE remained significant at 39% (95% CI 13%–58%). For PPSV23, 76% of cases and 78% of controls were vaccinated (*P*-value = .58), and there was no statistically significant VE in unadjusted [VE 11% (95% CI –27% to 37%)] or adjusted [–3.7% (95% CI –57% to 32%)] analysis. The receipt of both PCV13 and PPSV23 vaccination (39% in controls and 48% in cases) was associated with significant protection with adjusted VE of 39% (95% CI 12%–58%).

**Table 2:** Vaccine effectiveness against *Streptococcus pneumoniae* stratified by eGFR using a test-negative design^a^.

	Test-negative cases vaccinated (%)	Test-negative controls vaccinated (%)	Unadjusted VE	Adjusted VE
PCV13				
All cases	75/180 (42)	1982/3887 (51)	31 (7.2–49)	39 (13–58)
By eGFR:				
eGFR^b^ ≥60	44/117 (38)	1113/2495 (45)	25 (–9.2 to 49)	38 (2.9–61)
eGFR 30–59	26/55 (47)	709/1127 63)	47 (9.0–70)	61 (24–80)
eGFR <30	5/8 (62)	160/265 (60)		
PPSV23				
All cases	136/180 (76)	3018/3887 (78)	11 (–27 to 37)	–3.7 (–57 to 32)
By eGFR:				
eGFR ≥60	85/117 (73)	1853/2495 (74)	8.0 (–41 to 39)	–39 (–121 to 12)
eGFR 30–59	43/55 (78)	947/1127 (84)	32 (–37 to 64)	50 (1.9–75)
eGFR <30	8/8 (100)	218/265 (82)		
PCV13 and PPSV23				
All cases	70/180 (39)	1858/3887 (48)	31 (5.9–49)	39 (12–58)
By eGFR:				
eGFR ≥60	41/117 (35)	1043/2495 (42)	25 (–10 to 50)	38 (1.4–61)
eGFR 30–59	24/55 (44)	664/1127 (59)	46 (7.1–69)	58 (21–78)
eGFR <30	5/8 (62)	151/265 (57)		

a Adjusted VE was calculated by combining logistic regression for vaccine receipt between cases/controls and IPTW of overall vaccine receipt (i.e. doubly robust estimation). Covariates were identical for both and included demographics, medical comorbidities, eGFR and immunosuppression use. VE = 1 – OR × 100%.

b Race-free 2021 eGFR_Cr_ (mL/min/1.73 m^2^).

### Effectiveness of PCV13 and PPSV23 by eGFR strata

PCV13 vaccination uptake was lowest in those with eGFR ≥60 mL/min/1.73 m^2^ at 44%, and vaccination uptake in eGFR 30–59 and eGFR <30 mL/min/1.73 m^2^ strata were similar at 62% and 60%, respectively. After adjustment, there was significant PCV13 VE in both eGFR ≥60 mL/min/1.73 m^2^ [VE 38% (95% CI 2.9%–61%)] and eGFR 30–59 mL/min/1.73 m^2^ [VE 61% (95% CI 24%–80%), Table 2] without significant interaction between eGFR group and VE. Due to the small number of events, VE for eGFR <30 mL/min/1.73 m^2^ could not be calculated.

For PPSV23, vaccination uptake was lower in those with eGFR ≥60 mL/min/1.73 m^2^ at 74% compared with 84% in eGFR 30–60 and 83% in eGFR ≤30 mL/min/1.73 m^2^. The unadjusted VEs were 8.0% (95% CI –41% to 39%) in eGFR ≥60 mL/min/1.73 m^2^ and 32% (95% CI –37% to 64%) in eGFR 30–59 mL/min/1.73 m^2^. After adjustment for confounders, there was significant VE among eGFR 30–59 mL/min/1.73 m^2^ at 50% (95% CI 1.9%–75%, Table 2).

For individuals with both PCV13 and PPCV23 (41% in eGFR ≥60 mL/min/1.73 m^2^, and 58%/57%, respectively, in eGFR 30–59/<30 mL/min/1.73 m^2^), the adjusted VE in eGFR ≥60 mL/min/1.73 m^2^ was 38% (95% CI 1.4%–61%) and in eGFR 30–59 mL/min/1.73 m^2^ was 58% (95% CI 21%–78%, Table 2).

### Sensitivity analysis

In 21 bacteremic, test-negative cases (12%), we did not see significant PCV13 VE or PPSV23 VE, but the point estimates were similar to the primary analysis. In those with both PCV13 and PPSV23 vaccination, VE was statistically significant at 75% (95% CI 2.2%–94%). For non-bacteremic infection, PCV13 and both PCV13 and PPSV23 were associated with significant protection (Table 3). Non-bacteremic trends were similar across further stratification by eGFR ≥60 and 30–59 mL/min/1.73 m^2^ (Supplementary data, Table S3). When individuals were stratified by years since vaccination, the VEs were overall consistent without significant evidence of vaccine waning up to 5 years and 10 years after vaccination with PCV13 and PPSV23, respectively, but our sample size was limited (Table 4).

**Table 3:** Vaccine effectiveness against bacteremic and non-bacteremic *Streptococcus pneumoniae* using a test-negative design.

	Test-negative cases vaccinated (%)	Test-negative controls vaccinated (%)	Unadjusted VE	Adjusted VE
All cases				
PCV13	75/180 (42)	1982/3887 (51)	31 (7.2–49)	39 (13–58)
PPSV23	136/180 (76)	3018/3887 (78)	11 (–27 to 37)	–3.7 (–57 to 32)
PCV13 and PPSV23	70/180 (39)	1858/3887 (48)	31 (5.9–49)	39 (12–58)
Bacteremic cases				
PCV13	4/21 (19)	1982/3887 (51)	77 (39–94)	66 (–26 to 91)
PPSV23	15/21 (71)	3018/3887 (78)	28 (–102 to 71)	–19 (–226 to 57)
PCV13 and PPSV23	3/21 (14)	1858/3887 (48)	82 (46–96)	75 (2.2–94)
Nonbacteremic cases				
PCV13	71/159 (45)	1982/3887 (51)	23 (–6.6 to 44)	36 (5.4–56)
PPSV23	121/159 (76)	3018/3887 (78)	8.3 (–35 to 36)	–2.5 (–59 to 34)
PCV13 and PPSV23	67/159 (42)	1858/3887 (48)	21 (–9.4 to 43)	35 (3.2–56)

Adjusted VE was calculated by combining logistic regression for vaccine receipt between cases/controls and IPTW of overall vaccine receipt (i.e. doubly robust estimation). Covariates were identical for both and included demographics, medical comorbidities, eGFR, and immunosuppression use. VE = 1 – OR × 100%.

**Table 4:** Adjusted PCV13 and PPSV23 vaccine effectiveness by years since vaccination.

	Test-negative cases, *n*	Total , *n*	Unadjusted VE	Adjusted VE	*P*-value^a^
PCV13					
No vaccination	105	2035	0	0	.33
<2 years	28	782	29 (–6 to 55)	38 (–3.0 to 63)	
2–3 years	29	756	29 (–7 to 54)	38 (–0.5 to 62)	
4–5 years	14	436	44 (3.7–69)	42 (–19 to 71)	
>6 years	<10	58			
PPSV23					
No vaccination	46	947	0	0	.21
<5 years	77	2220	8.2 (–35 to 37)	–0.2 (–56 to 36)	
5–9 years	28	540	24 (–23 to 54)	17 (–45 to 52)	
10–14 years	20	259	4.8 (–61 to 46)	–37 (–162 to 28)	
>15 years	11	101	5.8 (–79 to 54)	–39 (–205 to 37)	

Adjusted VE was calculated by combining logistic regression for vaccine receipt between cases/controls and IPTW of overall vaccine receipt (i.e. doubly robust estimation). Vaccine receipt was stratified by time since vaccination compared with “no vaccination.” Covariates were identical for both models and included demographics, medical comorbidities, eGFR and immunosuppression use. VE = 1 – OR × 100%.

a *P*-value for linear trend.

Results were consistent when limiting controls to confirmed infection (Supplementary data, Table S4), restricting to individuals with a creatinine measurement in the previous 12 months (*N* = 3804), and redefining vaccination at ≥14 days prior to hospitalization. When excluding test-negative cases by ICD code (*N* < 10), PCV13 and combination PCV13 & PPSV23 were no longer significant in eGFR ≥60 [VE 36% (95% CI –1% to 59%) and VE 35% (95% CI –2.4% to 59%) respectively]. When we included PPSV23 receipt in PCV13 weighting and PCV13 receipt in PPSV23 weighting, the point estimates were also consistent, but there was significant covariate imbalance after IPTW due to the large overlap between PCV13 and PPSV23 recipients (Supplementary data, Fig. S4 and Table S5).

## DISCUSSION

In this test-negative design study using data from an integrated health system, we show that PCV13 confers significant VE against streptococcal pneumonia after accounting for underlying risk factors and probability of receiving vaccination. For PPSV23, VE was not evident. When stratified by eGFR, PCV13 protection was consistent in both eGFR ≥60 and 30–59 mL/min/1.73 m^2^, and findings were consistent in a series of sensitivity analyses which included stratification by years since vaccination, bacteremic infection and limiting controls to confirmed infections caused by other organisms. Taken together, our study demonstrated robust effectiveness of PCV13 in individuals with and without reduced eGFR, suggesting that further vaccination efforts with pneumococcal conjugate vaccines are warranted.

In our study, PCV13 population VE was 39% (95% CI 13%–58%) against pneumococcal pneumonia. Previous studies on PCV13 VE have mainly been from non-US countries with VE estimates between 33% and 45% [24, 26], which may have limited applicability to US populations due to variations in comorbid conditions, age, vaccination uptake and serotype prevalence. Specifically within the USA, prior studies have been limited by non-pathogen-specific pneumonia hospitalization [12, 27] or case–control design [28, 29]. One other US study has used a test-negative design, and PCV13 VE was 71% (95% CI 6%–91%) in individuals aged ≥65 years [30]. This VE was higher than ours, which could be related to the timing (immediately following the introduction of PCV13), lower prevalence of pneumococcal vaccination and serotype-matched design.

For PPSV23, we did not observe significant VE. This is not surprising as studies on PPSV23 efficacy [31, 32] have been inconsistent [33–35]. Explanations have included increased prevalence of immunization [36], herd immunity [37], changing serotype prevalence [31, 38], lower vaccination response [39] and waning after immunization [40]. Despite these conflicting data, PPSV23 vaccination remains important, especially in individuals with bacteremic disease [34] and in high-risk subgroups [41]. Pending changes in serotype prevalence and vaccination coverage, PPSV23 should be used as recommended given its expanded serotype coverage beyond certain conjugated vaccines [42].

To our knowledge, this is the first study to report streptococcal pneumococcal VE in a non-dialysis population with reduced eGFR. Prior studies of PCV in CKD have been limited to serologic data, and the implications of diminished immune responses on clinical outcomes has been unknown [43]. We show significant clinical benefit to vaccination in eGFR 30–59 mL/min/1.73 m^2^ with PCV13 VE 61% (95% CI 25%–80%). Interestingly, VE was higher in those with eGFR 30–59 than eGFR ≥60 mL/min/1.73 m^2^; this is unexpected as European CKD data show a higher prevalence of non-vaccine serotypes [44–46] which should result in lower VE. While we might observe this finding by chance, one hypothesis is that US patients with reduced eGFR have a higher prevalence of vaccine-matched strains due to differences in demographics, time since vaccination [47], antibiotic resistance patterns [48] and national vaccination programs [46, 49], but our observation requires further investigation. Regardless, our findings provide reassuring evidence on the clinical benefit of pneumococcal vaccination in individuals with reduced eGFR despite diminished immune response to vaccination [43, 50].

Our study has important clinical implications. In our test-negative study, 62% of individuals with eGFR <60 received PCV13 vaccination and 84% for PPSV23. Although these should not be extrapolated to the average population with reduced eGFR, our crude population prevalence was even lower, which raises concerns over missed opportunities to prevent pneumococcal disease and all-cause pneumonia [12]. The current ACIP guidelines recommend pneumococcal vaccination for individuals with chronic renal failure, but historically, chronic renal failure refers to those on dialysis which creates ambiguity in less severe CKD [4]. Furthermore, KDIGO recommends pneumococcal vaccination for eGFR <30 or eGFR 30–59 mL/min/1.73 m^2^ with certain medical conditions [5]. Thus, there is no agreement on pneumococcal vaccination for eGFR 30–59 mL/min/1.73 m^2^. This is an important issue because individuals with eGFR 30–59 mL/min/1.73 m^2^ comprise 93% of the US CKD population [2] and have a 40% increased risk of pneumonia hospitalization compared with those with eGFR >90 mL/min/1.73 m^2^ [3]. Pending further VE and serotype data, updated guidelines and new initiatives may be needed to increase pneumococcal vaccination uptake in those with CKD.

This study has limitations. First, urine antigen testing can have high specificity but low sensitivity [17] so we may have misclassified controls despite our case definition. Second, we did not have serotype matching, but our results likely reflect real-world effectiveness as serotype testing is not commonly performed in clinical practice. Third, generalizability is limited as this population was 97% white with recent outpatient creatinine measurement. Fourth, we were unable to assess chronicity of decreased eGFR. Fifth, we had limited power in the eGFR <30 mL/min/1.73 m^2^ strata, which requires furthers investigation as diminished VE has been observed in this group [51]. Furthermore, the rates of vaccination in this stratum (although a small sample) were higher among cases than controls—which would be consistent with diminished VE. Sixth, we were unable to isolate PCV13 effectiveness due to small number who received PCV13 alone, but point estimates were similar when we adjusted for history of PPSV23 vaccination. Also, the most recent ACIP guidelines now recommend PCV15 or PCV20 for PCV-naïve adults, but we would expect VE to be similar to or stronger than PCV13 given expanded serotype coverage. Seventh, 18% of vaccinations were by self-report. Eighth, although outcomes such as intensive care unit stay and mortality following pneumonia are important measures, our study was not powered enough to incorporate the severity of pneumonia. Finally, observational studies are limited by residual confounding, and we were unable to account for individual provider threshold for urine testing. Strengths of this study include the test-negative design and doubly robust VE estimation.

In conclusion, we found that PCV13 confers significant protection against non-serotyped *S. pneumoniae* hospitalization in individuals with eGFR 30–59 mL/min/1.73 m^2^ using a test-negative design. Efforts to increase pneumococcal conjugated vaccination in individuals with reduced eGFR are warranted.

## Supplementary Material

sfae145_Supplemental_File

## Data Availability

The data underlying this article will be shared on reasonable request to the corresponding author.

## Notes

Study funders did not have any role in study design, collection, analysis, interpretation of data, writing of the report or decision for submission for publication.
