# Supplementary material for: Pneumococcal vaccination effectiveness (PCV13 and PPSV23) in individuals with and without reduced kidney function: a test-negative design study
Source: Clin Kidney J. 2024 May 8;17(6):sfae145. doi: 10.1093/ckj/sfae145 (PMC11194481; doi:10.1093/ckj/sfae145)
Supplement: sfae145_Supplemental_File [file sfae145_supplemental_file.docx]

Supplement: Table of Contents

Tables:

Supplement Table 1. ICD-10 Diagnostic Codes

Supplement Table 2. Baseline Characteristics of the Overall Study Population by eGFR^a^ and Case Status^b^

Supplement Table 3. VE against Bacteremic and Nonbacteremic Streptococcal Pneumonia Stratified by eGFR^a^

Supplement Table 4. VE against *S. Pneumonia* Stratified by eGFR Compared to Controls with Speciated Infection By Culture or ICD-10 Code

Supplement Table 5. VE against *S. Pneumonia* by eGFR Accounting for Receipt of Other Pneumococcal Vaccinations

Supplement Table 6. Controls with Speciated Infection By Culture or ICD-10 Code

Figures:

Supplement Figure 1: Selection of Eligible Cases and Controls from all Hospitalizations in the Geisinger Health System

Supplement Figure 2: Crude PCV13 and PPSV23 Vaccination Prevalence from 2015 – 2021 Among Adults In Geisinger Health by Age > 18 – 64 or >65

Supplement Figure 3. SMD Before and After IPTW By Vaccination Status

Supplement Figure 4. SMD Before and After IPTW By Vaccination Status Including Other Pneumococcal Vaccination History

Supplement Table 1. ICD-10 Diagnostic Codes

| Diagnosis | ICD-10 Diagnostic Code |
| --- | --- |
| Myocardial infarction | I21.x, I22.x, I25.2 |
| Congestive heart failure | I09.9, I11.0, I13.0, I13.2, I25.5, I42.0, I42.5–I42.9, I43.x, I50.x, P29.0 |
| Peripheral vascular disease | I70.x, I71.x, I73.1, I73.8, I73.9, I77.1, I79.0, I79.2, K55.1, K55.8, K55.9, Z95.8, Z95.9 |
| Cerebrovascular disease | G45.x, G46.x, H34.0, I60.x–I69.x |
| Dementia | F00.x–F03.x, F05.1, G30.x, G31.1 |
| Respiratory disease | I27.8, I27.9, J40.x–J47.x, J60.x–J67.x, J68.4, J70.1, J70.3 |
| Rheumatic disease | M05.x, M06.x, M31.5, M32.x–M34.x, M35.1, M35.3, M36.0 |
| Peptic ulcer disease | K25.x–K28.x |
| Diabetes | E10.x–E14.x |
| Hemiplegia or paraplegia | G04.1, G11.4, G80.1, G80.2, G81.x, G82.x, G83.0–G83.4, G83.9 |
| Any malignancy, including lymphoma, leukemia and metastatic solid tumor, except malignant neoplasm of skin | C00.x–C26.x, C30.x–C34.x, C37.x–C41.x, C43.x, C45.x–C58.x, C60.x–C76.x, C77.x–C80.x, C81.x–C85.x, C88.x, C90.x–C97.x, |
| Liver Disease | B18.x, K70.0–K70.3, K70.9, K71.3–K71.5, K71.7, K73.x, K74.x, K76.0, K76.2–K76.4, K76.8, K76.9, Z94.4, I85.0, I85.9, I86.4, I98.2, K70.4, K71.1, K72.1, K72.9, K76.5, K76.6, K76.7 |

Supplement Table 2. Baseline Characteristics of the Overall Study Population by eGFR^*^ and Case Status^†^

| **eGFR Group** | **eGFR** ≥**60** | | | **eGFR 30-59** | | | **eGFR** ≥**15 & <30** | | |
| --- | --- | --- | --- | --- | --- | --- | --- | --- | --- |
|  | All N = 2,612 | Test-Negative  Cases  N = 117 | Test-Negative  Controls  N = 2,495 | All | Test-Negative  Cases  N = 55 | Test-Negative Controls  N = 1,127 | All | Test-Negative Cases | Test-Negative  Controls |
|  |  |  |  | N = 1,182 |  |  | N = 241 | N = 8 | N = 233 |
| eGFR | 87 (18) ^§^ | 86 (17) | 87 (18) | 47 (8) | 44 (9) | 47 (8) | 24 (4) | 25 (4) | 24 (4) |
| Age | 65 (16)^§^ | 64 (14) | 65 (16) | 77 (11%) | 75 (13) | 77 (11) | 77 (11) | 72 (15) | 77 (11) |
| Female | 1,177 (45%)^§^ | 70 (60%)^‡^ | 1,107 (44%) | 611 (52%) | 37 (67%)^‡^ | 574 (51%) | 137 (57%) | 4 (50%) | 133 (57%) |
| White | 2,524 (97%) | 111 (95%) | 2,413 (97%) | 1,157 (98%) | 55 (100%) | 1,102 (98%) | 235 (98%) | 8 (100%) | 227 (97%) |
| Body-Mass-Index | 29 (9) | 29 (9) | 29 (9) | 29 (9) | 30 (8) | 29 (9) | 30 (10) | 29 (6) | 30 (10) |
| Former Smoker | 1,244 (47%)^§^ | 55 (47%)^‡^ | 1,197 (48%) | 659 (56%) | 24 (43%)^‡^ | 635 (56%) | 120 (49%) | <10 | - |
| Current Smoker | 581 (22%)^§^ | 39 (33%)^‡^ | 549 (22%) | 109 (9.2%) | 12 (22%)^‡^ | 97 (8.6%) | 24 (10%) | <10 | - |
| PPSV23 | 1,938 (74%)^§^ | 85 (73%) | 1,853 (74%) | 990 (84%) | 43 (78%) | 947 (84%) | 199 (83%) | 8 (100%) | 191 (82%) |
| PCV13 | 1,157 (44%)^§^ | 44 (38%) | 1,113 (45%) | 735 (62%) | 26 (47%)^‡^ | 709 (63%) | 147 (61%) | <10 | - |
| PCV13 & PSPV23 | 1,084 (42%)^§^ | 41 (35%) | 1,043 (42%) | 688 (58%) | 24 (44%)^‡^ | 664 (59%) | 139 (58%) | <10 | - |
| Diabetes Mellitus | 563 (22%)^§^ | 23 (20%) | 540 (22%) | 455 (39%) | 21 (38%) | 434 (39%) | 131 (54%) | <10 | - |
| Myocardial Infarction | 79 (3%)^§^ | <10 (2%) | - | 66 (6%) | <10 (9.1%) | - | 18 (8%) | <10 | - |
| Stroke | 189 (7%)^§^ | 10 (8.5%) | 179 (7.2%) | 131 (11%) | <10 (16%) | - | 27 (11%) | <10 | - |
| CHF | 350 (13%)^§^ | 20 (17%) | 330 (13%) | 383 (32%) | 14 (26%) | 369 (33%) | 103 (43%) | <10 | - |
| PVD | 183 (7%)^§^ | 12 (10%) | 171 (6.9%) | 158 (13%) | <10 (13%) | - | 41 (17%) | 0 (0.0%) | 41 (18%) |
| Dementia | 27 (1%) | <10 (2%) | - | 21 (2%) | <10 (2%) | - | <10 (0%) | 0 (0.0%) | <10 (0.4%) |
| Respiratory | 926 (36%) | 43 (37%) | 883 (35%) | 409 (35%) | 21 (38%) | 388 (34%) | 66 (27%) | <10 | - |
| COPD | 697 (27%) | 35 (30%) | 662 (27%) | 320 (27%) | 19 (35%) | 301 (27%) | 52 (22%) | <10 | - |
| Rheumatologic | 76 (3%) | <10 (3%) | - | 35 (3%) | <10 (4%) | - | <10 (3%) | 0 (0.0%) | <10 (3%) |
| Cancer | 377 (14%) | 16 (14%) | 361 (15%) | 133 (11%) | <10 (11%) | - | 32 (13%) | <10 | - |
| Liver | 100 (4%) | <10 (3%) | - | 22 (2%) | <10 (4%) | - | <10 (3%) | <10 | - |
| Immunosuppression | 121 (5%) | <10 (6%) | - | 39 (3%) | <10 (4%) | - | <10 (4%) | <10 | - |
| Peptic Ulcer Disease | <10 (0%) | <10 (1%) | - | <10 (0%) | <10 (4%) | - | <10 (0%) | 0 (0.0%) | <10 (0.4%) |
| Hemiplegia | 15 (1%) | 0 (0.0%) | 15 (0.6%) | <10 (0%) | 0 (0.0%) | - | <10 (0%) | 0 (0.0%) | 0 (0.0%) |
| Hospitalization (<1 yr) | 1,617 (62%) | 69 (59%) | 1548 (62%) | 734 (62%) | 31 (56%) | 703 (62%) | 148 (61%) | <10 | 143 (61%) |

* Race-free 2021 eGFR_Cr_ presented as mean (standard deviation) and measured as mL/min/1.73 m^2^

^†^ Data are presented as mean (standard deviation) or mean (percent)

^‡^ P-value <0.05 between Cases and Controls within eGFR category by Pearson χ2, fisher’s exact (N < 10), or t tests Comparing Cases vs Controls

^§^ P-value <0.01 across all eGFR categories of ≥60, 30-59, and <30

Abbreviations: PPSV23, Pneumococcal polysaccharide vaccine (PPSV23); PCV13, Pneumococcal conjugate vaccine (PCV13); CHF, Congestive heart failure; PVD, peripheral vascular disease; COPD, Chronic obstructive pulmonary disease; eGFR, estimated glomerular rate

Supplement Table 3. VE against Bacteremic and Nonbacteremic Streptococcal Pneumonia Stratified by eGFR

|  | Test-Negative Cases  Vaccinated (%) | | Test-Negative Controls  Vaccinated (%) | | Unadjusted VE | Adjusted VE |
| --- | --- | --- | --- | --- | --- | --- |
| PCV13 Bacteremic | 4/21 | (19%) | 1,982/3,887 | (51%) | 77 (39 – 94) | 66 (-26 – 91) |
| By eGFR: |  |  |  |  |  |  |
| eGFR^†^ ≥60 | 4/19 | (21%) | 1113/2495 | (45%) | 67 (8.5 – 91) | - |
| eGFR 30 – 59 | 0/2 | (0%) | 709/1127 | (63%) | - | - |
| eGFR <30 | 0 |  | 160/265 | (60%) | - | - |
| PPSV23 Bacteremic | 15/21 | (71%) | 3,018/3,887 | (78%) | 28 (-102 – 71) | -19 (-226 – 57) |
| By eGFR: |  |  |  |  |  |  |
| eGFR ≥60 | 13/19 | (68%) | 1853/2495 | (74%) | 25 (-114 – 71) | - |
| eGFR 30 – 59 | 2/2 | (100%) | 947/1127 | (84%) | - | - |
| eGFR <30 | 0 |  | 160/265 | (60%) | - | - |
| PCV13 & PPSV23 Bacteremic | 3/21 | (14%) | 1,858/3,887 | (48%) | 82 (46 – 96) | 75 (2.2 – 94) |
| By eGFR: |  |  |  |  |  |  |
| eGFR ≥60 | 3/19 | (16%) | 1043/2495 | (42%) | 74 (21 – 94) | - |
| eGFR 30 – 59 | 0/2 | (0%) | 664/1127 | (59%) | - | - |
| eGFR <30 | 0 |  | 160/265 | (60%) | - | - |
|  | Test-Negative Cases  Vaccinated (%) | | Test-Negative Controls  Vaccinated (%) | | Unadjusted VE | Adjusted VE |
| PCV13 Nonbacteremic | 71/159 | (45%) | 1,982/3,887 | (51%) | 23 (-6.6 – 44) | 36 (5.4– 56) |
| By eGFR: |  |  |  |  |  |  |
| eGFR ≥60 | 40/98 | (41%) | 1113/2495 | (45%) | 14 (-29 – 44) | 32 (-9.2 – 58) |
| eGFR 30 – 59 | 26/53 | (49%) | 709/1127 | (63%) | 43 (1.2 – 67) | 59 (20 – 79) |
| eGFR <30 | 5/8 | (62%) | 160/265 | (60%) | - | - |
| PPSV23 Nonbacteremic | 121/159 | (76%) | 3,018/3,887 | (78%) | 8.3 (-35 – 36) | -2.5 (-59 – 34) |
| By eGFR: |  |  |  |  |  |  |
| eGFR ≥60 | 72/98 | (73%) | 1853/2495 | (74%) | 4.1 (-54 – 39) | -47 (-144 – 12) |
| eGFR 30 – 59 | 41/53 | (77%) | 947/1127 | (84%) | 35 (-31 – 66) | 53 (6.5 – 76) |
| eGFR <30 | 8/8 | (100%) | 218/265 | (82%) | - | - |
| PCV13 & PPSV23 Nonbacteremic | 67/159 | (42%) | 1,858/3,887 | (48%) | 21 (-9.4 – 43) | 35 (3.2 – 56) |
| By eGFR: |  |  |  |  |  |  |
| eGFR ≥60 | 38/98 | (39%) | 1043/2495 | (42%) | 12 (-33 – 42) | 29 (-15 – 56) |
| eGFR 30 – 59 | 24/53 | (45%) | 664/1127 | (59%) | 42 (-0.3 – 67) | 57 (16 – 78) |
| eGFR <30 | 5/8 | (62%) | 151/265 | (57%) | – | – |

Adjusted VE calculated by combining logistic regression for vaccine receipt between cases/controls after IPTW of overall vaccine receipt (doubly robust estimation). Covariates were identical for both and included demographics, medical comorbidities, eGFR, and immunosuppression use. VE = (1-OR) x 100%.

† Race-free 2021 eGFR_Cr_ (mL/min/1.73 m^2^)

Abbreviations: PCV13, Pneumococcal conjugate vaccine (PCV13); PPSV23, Pneumococcal polysaccharide vaccine (PPSV23); eGFR, estimated glomerular function; IPTW, inverse probability of treatment weighting; VE, vaccine effectiveness

Supplement Table 4. VE against *S. Pneumonia* Stratified by eGFR Compared to Controls with Speciated Infection By Culture or ICD-10 Code

|  | Test-Negative Cases  Vaccinated (%) | | Test-Negative Controls  Vaccinated (%) | | Unadjusted VE | Adjusted VE^†^ |
| --- | --- | --- | --- | --- | --- | --- |
| PCV13–All | 75/180 | (42%) | 363/683 | (53%) | 37 (12 – 55) | 40 (14 – 58) |
| By eGFR: |  |  |  |  |  |  |
| eGFRb ≥60 | 44/117 | (38%) | 202/450 | (45%) | 26 (-12 – 52) | 38 (3.2 – 61) |
| eGFR 30 – 59 | 26/55 | (47%) | 131/191 | (69%) | 59 (24 – 78) | 62 (26 – 81) |
| eGFR <30 | 5/8 | (62%) | 30/42 | (71%) | - | - |
| PPSV23–All | 136/180 | (76%) | 529/683 | (77%) | 10 (-33 – 38) | -3.7 (-57 – 32) |
| By eGFR: |  |  |  |  |  |  |
| eGFR ≥60 | 85/117 | (73%) | 328/450 | (73%) | 1. (-58 – 37) | -39 (-120 – 13) |
| eGFR ≥30 & <60 | 43/55 | (78%) | 166/191 | (87%) | 46 (-19 – 75) | 51 (3.8 – 75) |
| eGFR <30 | 8/8 | (100%) | 35/42 | (83%) | - | - |
| PCV13 & PPSV23–All | 70/180 | (39%) | 344/683 | (50%) | 31 (6 – 49) | 41 (14 – 59) |
| By eGFR: |  |  |  |  |  |  |
| eGFR ≥60 | 41/117 | (35%) | 190/450 | (42%) | 26 (-12 – 52) | 38 (2.1 – 61) |
| eGFR 30 – 59 | 24/55 | (44%) | 124/191 | (65%) | 61 (23 – 80) | 60 (23 – 79) |
| eGFR <30 | 5/8 | (62%) | 30/42 | (71%) | - | – |
|  | Vaccinated Case | | Vaccinated Controls | | Unadjusted VE | Adjusted VE |
| PCV13 Bacteremic | 4/21 | (19%) | 363/683 | (53%) | 79 (43 – 94) | 63 (-18 – 88) |
| By eGFR: |  |  |  |  |  |  |
| eGFR ≥60 | 4/19 | (21%) | 202/450 | (45%) | 67 (8 – 91) | - |
| eGFR 30 – 59 | 0/2 | (0%) | 131/191 | (69%) | - | - |
| eGFR <30 | 0 |  | 30/42 | (71%) | - | - |
| PPSV23 Bacteremic | 15/21 | (71%) | 529/683 | (77%) | 27 (-107 – 71) | -8.1 (-266 – 68) |
| By eGFR: |  |  |  |  |  |  |
| eGFR ≥60 | 13/19 | (68%) | 328/450 | (73%) | 19 (-134 – 69) | - |
| eGFR 30 – 59 | 2/2 | (100%) | 166/191 | (87%) | - | - |
| eGFR <30 | 0 |  | 35/42 | (83%) | - | - |
| PCV13 & PPSV23 Bacteremic | 3/21 | (14%) | 344/683 | (50%) | 84 (51 – 96) | 56 (-32 – 86) |
| By eGFR: |  |  |  |  |  |  |
| eGFR ≥60 | 3/19 | (16%) | 190/450 | (42%) | 74 (27 – 94) | - |
| eGFR 30 – 59 | 0/2 | (0%) | 124/191 | (65%) | - | - |
| eGFR <30 | 0 |  | 30/42 | (71%) | - | - |
|  | Vaccinated Case | | Vaccinated Controls | | Unadjusted VE | Adjusted VE |
| PCV13 Nonbacteremic | 71/159 | (45%) | 363/683 | (53%) | 29 (-0.5 – 50) | 46 (17 – 65) |
| By eGFR: |  |  |  |  |  |  |
| eGFR ≥60 | 40/98 | (41%) | 202/450 | (45%) | 15 (-32 – 46) | 41 (-1.7 – 66) |
| eGFR 30 – 59 | 26/53 | (49%) | 131/191 | (69%) | 56 (18 – 76) | 70 (32 – 87) |
| eGFR <30 | 5/8 | (62%) | 30/42 | (71%) | - | - |
| PPSV23 Nonbacteremic | 121/159 | (76%) | 529/683 | (77%) | 7 (-41 – 38) | -11 (-89 – 34) |
| By eGFR: |  |  |  |  |  |  |
| eGFR ≥60 | 72/98 | (73%) | 328/450 | (73%) | -3 (-71 – 37) | -113 (-313 – 9.6) |
| eGFR 30 – 59 | 41/53 | (77%) | 166/191 | (87%) | 49 (-14 – 76) | 63 (9.7 – 85) |
| eGFR <30 | 8/8 | (100%) | 35/42 | (83%) | - | - |
| PCV13 & PPSV23 Nonbacteremic | 67/159 | (42%) | 344/683 | (50%) | 28 (-2 – 50) | 38 (1.4 – 61) |
| By eGFR: |  |  |  |  |  |  |
| eGFR ≥60 | 38/98 | (39%) | 190/450 | (42%) | 13 (-35 – 45) | 32 (-19 – 61) |
| eGFR 30 – 59 | 24/53 | (45%) | 124/191 | (65%) | 55 (17 – 76) | 70 (31 – 87) |
| eGFR <30 | 5/8 | (62%) | 30/42 | (71%) | - | – |

Adjusted VE calculated by combining logistic regression for vaccine receipt between cases/controls after IPTW of overall vaccine receipt (doubly robust estimation). Covariates were identical for both and included demographics, medical comorbidities, eGFR, and immunosuppression use. VE = (1-OR) x 100%.

† Race-free 2021 eGFR_Cr_ (mL/min/1.73 m^2^)

Abbreviations: PCV13, Pneumococcal conjugate vaccine (PCV13); PPSV23, Pneumococcal polysaccharide vaccine (PPSV23); eGFR, estimated glomerular function; IPTW, inverse probability of treatment weighting; VE, vaccine effectiveness

Supplement Table 5. VE against *S. Pneumonia* Stratified by eGFR Accounting for Receipt of Other Pneumococcal Vaccinations

|  | Vaccinated Cases | | Vaccinated Controls | | Unadjusted VE | Adjusted VE^†^ |
| --- | --- | --- | --- | --- | --- | --- |
| PCV13–All | 75/180 | (42%) | 1,982/3,887 | (51%) | 31 (7.2 – 49) | 36 (1.7 – 58) |
| By eGFR: |  |  |  |  |  |  |
| eGFR ≥60 | 44/117 | (38%) | 1,113/2,495 | (45%) | 25 (-9.2 – 49) | 38 (-7.0 – 64) |
| eGFR 30 – 59 | 26/55 | (47%) | 709/1,127 | (63%) | 47 (9.0 – 70) | 58 (13 – 79) |
| eGFR <30 | 5/8 | (62%) | 160/265 | (60%) | - | - |
| PPSV23–All | 136/180 | (76%) | 3,018/3,887 | (78%) | 11 (-27 – 37) | -13 (-87 – 32) |
| By eGFR: |  |  |  |  |  |  |
| eGFR ≥60 | 85/117 | (73%) | 1,853/2,495 | (74%) | 8.0 (-41 – 39) | -44 (-139 – 13) |
| eGFR ≥30 & <60 | 43/55 | (78%) | 947/1,127 | (84%) | 32 (-37 – 64) | 40 (-24 – 71) |
| eGFR <30 | 8/8 | (100%) | 218/265 | (82%) | - | - |
|  | Vaccinated Case | | Vaccinated Controls | | Unadjusted VE | Adjusted VE |
| PCV13 Bacteremic | 4/21 | (19%) | 1,982/3,887 | (51%) | 77 (39 – 94) | 47 (-171 – 90) |
| By eGFR: |  |  |  |  |  |  |
| eGFR ≥60 | 4/19 | (21%) | 1113/2495 | (45%) | 67 (8.5 – 91) | - |
| eGFR 30 – 59 | 0/2 | (0%) | 709/1127 | (63%) | - | - |
| eGFR <30 | 0 |  | 160/265 | (60%) | - | - |
| PPSV23 Bacteremic | 15/21 | (71%) | 3,018/3,887 | (78%) | 28 (-102 – 71) | -8.9 (-308 – 71) |
| By eGFR: |  |  |  |  |  |  |
| eGFR ≥60 | 13/19 | (68%) | 1853/2495 | (74%) | 25 (-114 – 71) | - |
| eGFR 30 – 59 | 2/2 | (100%) | 947/1127 | (84%) | - | - |
| eGFR <30 | 0 |  | 160/265 | (60%) | - | - |
|  | Vaccinated Case | | Vaccinated Controls | | Unadjusted VE | Adjusted VE |
| PCV13 Nonbacteremic | 71/159 | (45%) | 1,982/3,887 | (51%) | 23 (-6.6 – 44) | 42 (5.1 – 65) |
| By eGFR: |  |  |  |  |  |  |
| eGFR ≥60 | 40/98 | (41%) | 1113/2495 | (45%) | 14 (-29 – 44) | 50 (5.3 – 73) |
| eGFR 30 – 59 | 26/53 | (49%) | 709/1127 | (63%) | 43 (1.2 – 67) | 64 (15 – 85) |
| eGFR <30 | 5/8 | (62%) | 160/265 | (60%) | - | - |
| PPSV23 Nonbacteremic | 121/159 | (76%) | 3,018/3,887 | (78%) | 8.3 (-35 – 36) | -9.2 (-94 – 38) |
| By eGFR: |  |  |  |  |  |  |
| eGFR ≥60 | 72/98 | (73%) | 1853/2495 | (74%) | 4.1 (-54 – 39) | -111 (-300 – -11) |
| eGFR 30 – 59 | 41/53 | (77%) | 947/1127 | (84%) | 35 (-31 – 66) | 60 (-4.6 – 84) |
| eGFR <30 | 8/8 | (100%) | 218/265 | (82%) | - | - |

Adjusted VE calculated by combining logistic regression for vaccine receipt between cases/controls after IPTW of overall vaccine receipt (doubly robust estimation). Covariates were identical for both and included demographics, medical comorbidities, eGFR, and immunosuppression use. VE = (1-OR) x 100%.

† Race-free 2021 eGFR_Cr_ (mL/min/1.73 m^2^)

Abbreviations: PCV13, Pneumococcal conjugate vaccine (PCV13); PPSV23, Pneumococcal polysaccharide vaccine (PPSV23); eGFR, estimated glomerular function; IPTW, inverse probability of treatment weighting; VE, vaccine effectiveness

Supplement Table 6. Controls with Speciated Infection By Culture or ICD-10 Code

| Species | Culture or PCR | ICD-10 | Total |
| --- | --- | --- | --- |
| Adenovirus |  | 4 | 4 |
| Candida Species | 4 |  | 4 |
| Enterobacter Species | 5 |  | 5 |
| Enterococcus Species | 20 |  | 20 |
| Escherichia Coli | 37 | 3 | 40 |
| Haemophilis Influenza | 8 | 15 | 23 |
| Human Metapneumovirus |  | 38 | 38 |
| Influenza |  | 83 | 83 |
| Klebsiella Pneumoniae | 9 | 3 | 12 |
| Legionella pneumophila^*^ | 35 |  | 35 |
| Mycoplasma pneumoniae |  | 5 | 5 |
| Other gram negative pneumoniae |  | 88 | 88 |
| Parainfluenza |  | 12 | 12 |
| Proteus Species | 4 |  | 4 |
| Pseudomonas Aeruginosa | 6 | 24 | 30 |
| RSV |  | 19 | 19 |
| Serratia Marcescens | 3 |  | 3 |
| SARS-CoV-2^†^ | 81 |  | 81 |
| Staphylococcus Aureus | 105 | 36 | 141 |
| Streptococcus Species | 36 |  | 36 |
| Total | 354 | 330 | 683 |

*Urinary antigen test

† nasal swab

Abbreviations: SARS-CoV-2, severe acute respiratory syndrome coronavirus 2.

Supplement Table 7. VE against *S. Pneumonia* Adjusting for ICU Stay During Hospitalization

| PCV13 | Test-Negative Cases  Vaccinated (%) | | Test-Negative Controls  Vaccinated (%) | | Unadjusted VE | Adjusted VE |
| --- | --- | --- | --- | --- | --- | --- |
| All cases | 75/180 | (42%) | 1,982/3,887 | (51%) | 37 (8.3 – 57) | 39 (13 – 58) |
| By eGFR: |  |  |  |  |  |  |
| eGFR^†^ ≥60 | 44/117 | (38%) | 1,113/2,495 | (45%) | 37 (-0.1 – 60) | 38 (3.0 – 61) |
| eGFR 30 – 59 | 26/55 | (47%) | 709/1,127 | (63%) | 57 (17 – 78) | 61 (25 – 80) |
| eGFR <30 | 5/8 | (62%) | 160/265 | (60%) | - | - |
| PPSV23 |  | |  |  |  |  |
| All cases | 136/180 | (76%) | 3,018/3,887 | (78%) | -0.2 (-65 – 39) | -3.6 (-57 – 32) |
| By eGFR: |  |  |  |  |  |  |
| eGFR ≥60 | 85/117 | (73%) | 1,853/2,495 | (74%) | -44 (-128 – 9.4) | -41 (-123 – 11) |
| eGFR 30 – 59 | 43/55 | (78%) | 947/1,127 | (84%) | 60 (-7.1 – 85) | 51 (2.9 – 75) |
| eGFR <30 | 8/8 | (100%) | 218/265 | (82%) | - | - |
| PCV13 & PPSV23 |  | |  |  |  |  |
| All cases | 70/180 | (39%) | 1,858/3,887 | (48%) | 37 (6.3 – 58) | 40 (12 – 58) |
| By eGFR: |  |  |  |  |  |  |
| eGFR ≥60 | 41/117 | (35%) | 1,043/2,495 | (42%) | 36 (-2.7 – 60) | 38 (1.3 – 61) |
| eGFR 30 – 59 | 24/55 | (44%) | 664/1,127 | (59%) | 57 (19 – 78) | 59 (21 – 78) |
| eGFR <30 | 5/8 | (62%) | 151/265 | (57%) | – | – |

ICU care during the same hospitalization was included in the IPTW and regression model. VE = (1-OR) x 100%.

† Race-free 2021 eGFR_Cr_ (mL/min/1.73 m^2^)

Abbreviations: PCV13, Pneumococcal conjugate vaccine (PCV13); PPSV23, Pneumococcal polysaccharide vaccine (PPSV23); eGFR, estimated glomerular function; IPTW, inverse probability of treatment weighting; VE, vaccine effectiveness

Supplement Figure 1: Selection of Eligible Cases and Controls from all Hospitalizations in the Geisinger Health System


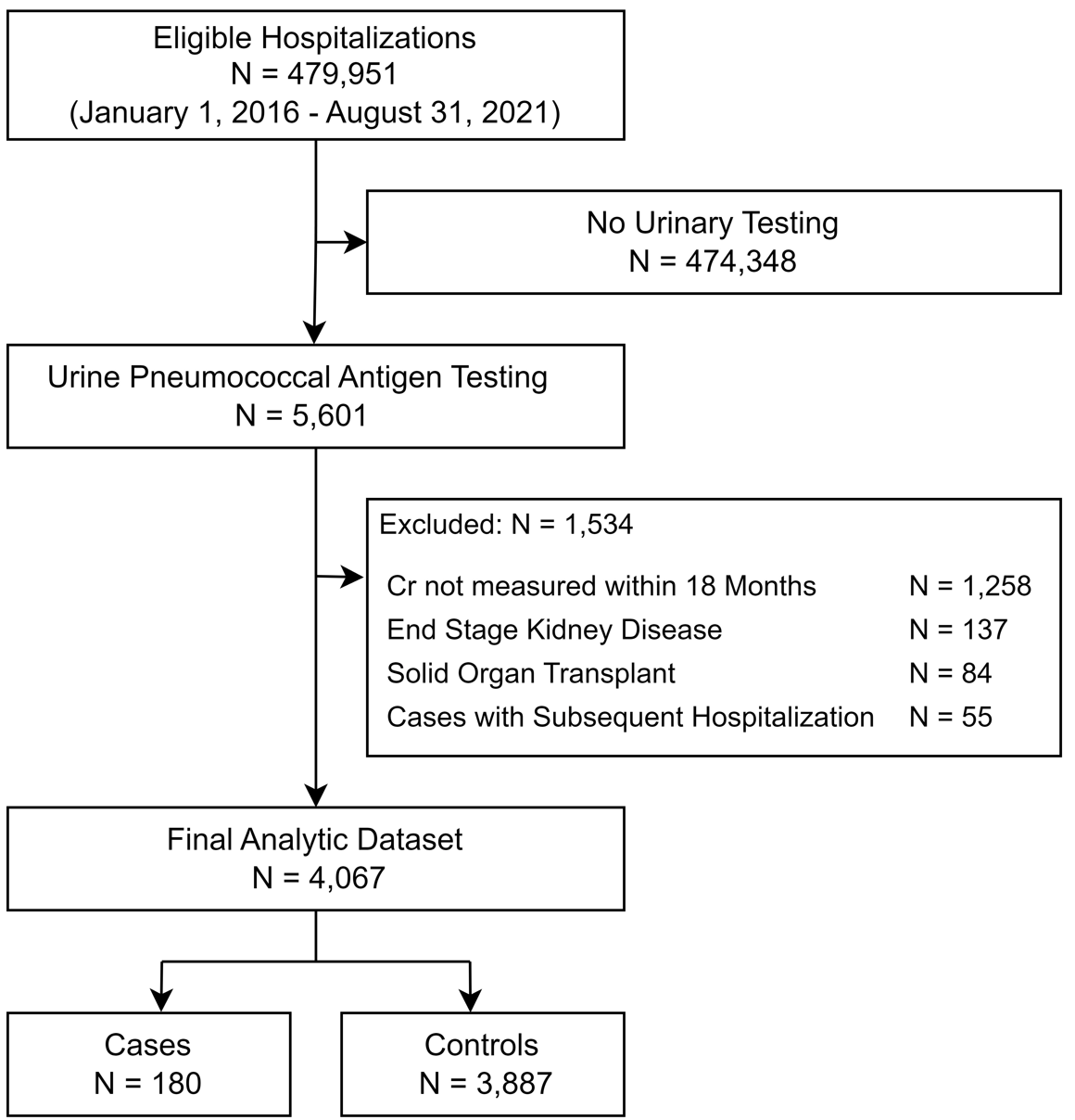


Selection criteria for this test-negative design study. All hospitalizations within the data range were eligible for inclusion. All cases and controls had a urinary streptococcal pneumonia test from the eligible hospitalization. Cases were defined as a positive urinary test, sterile culture, or ICD-10 code for streptococcal pneumonia. Transplant and ESKD status were defined by ICD-10 code.

Abbreviations: N, Number; Cr, Creatinine; ICD-10, International Classification of Disease – CM Code 10.

Supplement Figure 2. Crude PCV13 and PPSV23 Vaccination Prevalence from 2015 – 2021 Among Adults In Geisinger Health by Age > 18 – 64 (N = 302,726; top row) or >65 (N = 128,842; bottom row)


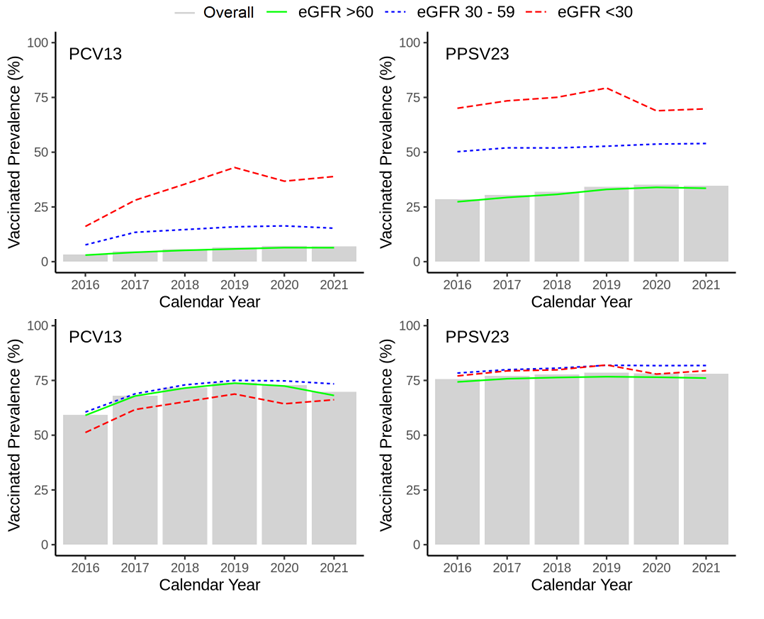


Supplement Figure 3. SMD Before and After IPTW By Vaccination Status

Compared to the 180 cases and 3887 controls, the post-IPTW populations were 174 cases and 4050 controls for PCV13, 174 cases and 3964 controls for PPSV23, and 164 cases and 3924 controls for PCV13 & PPSV23.

Supplement Figure 4. SMD Before and After IPTW By Vaccination Status Including Other Pneumococcal Vaccination History
